# Antidepressants and health-related quality of life (HRQoL) for patients with depression: Analysis of the medical expenditure panel survey from the United States

**DOI:** 10.1371/journal.pone.0265928

**Published:** 2022-04-20

**Authors:** Omar A. Almohammed, Abdulaziz A. Alsalem, Abdullah A. Almangour, Lama H. Alotaibi, Majed S. Al Yami, Leanne Lai

**Affiliations:** 1 Department of Clinical Pharmacy, College of Pharmacy, King Saud University, Riyadh, Saudi Arabia; 2 Pharmacoeconomics Research Unit, College of Pharmacy, King Saud University, Riyadh, Saudi Arabia; 3 Department of Pharmacy Practice, College of Pharmacy, King Saud bin Abdulaziz University for Health Sciences, Riyadh, Saudi Arabia; 4 Department of Clinical Pharmacy, King Abdulaziz Medical City, Riyadh, Saudi Arabia; 5 King Abdullah International Medical Research Center, Ministry of National Guard Health Affairs, Riyadh, Saudi Arabia; 6 Department of Sociobehavioral and Administrative Pharmacy, College of Pharmacy, Nova Southeastern University, Davie, Florida, United States of America; The University of Sydney, AUSTRALIA

## Abstract

**Background:**

Despite the empirical literature demonstrating the efficacy of antidepressant medications for treatment of depression disorder, these medications’ effect on patients’ overall well-being and health-related quality of life (HRQoL) remains controversial. This study investigates the effect of antidepressant medication use on patient-reported HRQoL for patients who have depression.

**Methods:**

A comparative cohort, secondary database analysis was conducted using data from the United States’ Medical Expenditures Panel Survey for patients who had depression. HRQoL was measured using the SF-12 and reported as physical and mental component summaries (PCS and MCS). A cohort of patients that used antidepressant medications were compared to a cohort of patients that did not. Univariate and multivariate difference-in-differences (D-I-D) analyses were used to assess the significance of the mean difference of change on the PCS and MCS from baseline to follow-up.

**Results:**

On average, 17.5 million adults were diagnosed with depression disorder each year during the period 2005–2016. The majority were female (67.9%), a larger proportion of whom received antidepressant medications (60.5% vs. 51.5% of males). Although use of antidepressants was associated with some improvement on the MCS, D-I-D univariate analysis revealed no significant difference between the two cohorts in PCS (–0.35 vs. –0.34, p = 0.9595) or MCS (1.28 vs. 1.13, p = 0.6405). The multivariate D-I-D analyses ensured the robustness of these results.

**Conclusion:**

The real-world effect of using antidepressant medications does not continue to improve patients’ HRQoL over time. Future studies should not only focus on the short-term effect of pharmacotherapy, it should rather investigate the long-term impact of pharmacological and non-pharmacological interventions on these patients’ HRQoL.

## Introduction

Approximately one out of every five adults (aged 18 or older) living in the United States (US) experience some type of mental illness every year [1], and depression is the most prevalent among others [2]. The National Institute of Mental Health (NIMH) estimated that about 17.3 million adults in the US had at least one major depressive episode in 2017 [1]. In addition, patients with one or more chronic condition(s) have an increased risk of developing major depression [3], which usually present as persistent sadness, lack of interest, poor concentration and change in mood [2].

Depression can negatively impact these patients’ health-related quality of life (HRQoL) and this can be linked to them having unhealthy or risky behaviors including inactivity, overeating, smoking, and noncompliance to their medications [3]. This effect was seen on the short form (36) health survey (SF-36), which indicated the significant impact for depression on patients’ HRQoL when patients with depression where compared to those without, especially on the mental component of the SF-36 [4]. Additionally, the presence of major depressive disorder (MDD) as a comorbid condition in patients with other medical condition leads to an even worse HRQoL compared to patients with the medical condition only. This was true among patients with diabetes [5–7], hypertension [7, 8], asthma [9], and cancer [10, 11]. These findings were important as it revealed a significant increase in the cost of care for the management of these comorbidities when MDD exist as a comorbid condition. Therefore, screening for and treating depression in these patients may improve their HRQoL and reduce the overall medical cost when managing patients with these conditions.

In general, depression disorder puts a huge burden on the global and national economy and it is steadily increasing. In the US, the cost of managing MDD alone was around US$ 66 billion in 2005 and this number increased by 21% in 2010 to reach US$ 80 billion [12]. A more recent estimation of total annual healthcare costs of managing mood disorders, including both depressive and bipolar disorders, shows that the health care costs for patients with mood disorders were more than double that of their counterpart without a mood disorder. The estimated individual cost was around US$ 6,591 annually (adjusted for inflation to 2017 USD); in total, the national annual direct incremental healthcare expenditures on mood disorders in the United States was averaging to $172 billion between 2007 and 2017. These figures were calculated after adjusting for sociodemographic factors and comorbidities and does not include intangible or indirect costs which are estimated to account for more than half the total economic burden of mood disorders [13]. Moreover, both direct and indirect costs per year were higher for patients with depression, especially for patients with treatment-resistant depression [14]. Therefore, the inadequate treatment of patients with depression can continuously increase the societal cost of their illness.

Among patients with depression, the use of psychotherapy and pharmacotherapy are efficacious in improving patients’ symptoms and quality of life. However, it is better to use the combination of the two treatment options as it has a better effect than using each option separately [15]. Although psychotherapy appears to be slightly more efficacious than pharmacotherapy, there is no strong evidence that one of the two treatment options are better than other [15]. However, the American Psychiatric Association (APA) guideline recommendations include the use of either psychotherapy or second-generation antidepressants, such as selective serotonin reuptake inhibitors (SSRIs) or serotonin-norepinephrine reuptake inhibitors (SNRIs), for adult patients as initial therapy. For patients who have a partial or no response to the initial therapy, the guideline recommend switching from pharmacotherapy alone to cognitive therapy alone or switch from one antidepressant medication to another antidepressant medication from the same or different class [16].

Published clinical trials have stated that the utilization of psychopharmacotherapy may improve the clinical outcomes of patients. However, the evidence around the beneficial overall effect for these medications in patients with depression disorder is controversial. Multiple meta-analyses were conducted to assess the change in outcomes for patients treated for MDD including different antidepressant medications. These studies concluded that most of the improvement on symptoms (about 80%) came from the placebo effect for these medications [17–19]. They found that the difference between the placebo and treatment groups was very minimal in the meta-analyses that included data from published studies [17–19] and when data from unpublished studies were combined with data from published studies the difference became statistically insignificant, or even clinically undetectable [17, 19]. Moreover, when psychotherapy was evaluated against antidepressants, it was found to have a comparable efficacy to prescribed antidepressants [17].

The controversial results in the clinical outcomes from the clinical trials and the meta-analyses of these trials need to be further evaluated to improve the care provided to these patients. Despite the empirical literature demonstrating the efficacy of these psychotherapeutic medications for depression disorder, the impact of these medications on patients’ overall well-being and quality of life remains controversial. The effect of the psychopharmacotherapy on patients’ overall heath has not been thoroughly evaluated using patient reported outcome (PRO) measures, such as the HRQoL. PRO measures are good indicators that can be utilized in this case because improving patients’ outcomes are the ultimate goal of therapy. The measures of HRQoL, such as the SF-36, have been proven to capture change in physical and mental functionality in many patients’ populations including patients with mental illness [20–24]. The aim of this study was to investigate the effect of using antidepressant medications on the HRQoL among patients with depression. This will shed the light on some of the current limitations in the care provided to patients with depression as well as highlight areas for improvement when providing care or evaluating new therapy for patients with depression.

## Methods

### Study design and data source

A comparative cohort, secondary database analysis was conducted using data from the Medical Expenditures Panel Survey (MEPS). In the United States, MEPS is a nationally representative database that uses a subsample of households that participated in the previous year’s National Health Interview survey (NHIS) conducted by the National Center for Health Statistics (NCHS). The MEPS utilize a longitudinal, complex, multistage sampling methodology with clustering and oversampling of certain groups, like minorities. Each year a new panel is introduced to the MEPS database, and five in-person computer-assisted personal interview are conducted over 30 months of follow-up to capture personal data for two calendar years for each person and family in the panel. The MEPS is based on utilizing self-reported data from families and individuals; however, all health care expenditure and utilization and employment data are verified through medical providers and employers across the US. After that, all respondents in the panel are assigned different types of weights (one of these weights is for the longitudinal two years follow-up data file) in order to permit generating national estimates on expenditure and utilization, health status, health insurance coverage, source of payment, and socio-demographic and socio-economic characteristics for the civilian, noninstitutionalized US population. Generally, every year the MEPS publish different types of data files derived from the data collected in the previous years, these include medical condition, prescribed medicines, employment variables, longitudinal data files, …etc. However, to meet the objective of the study, we utilized the two-year longitudinal data files for the panels 10 to 20 which include data that were collected between 2005 and 2016, along with the related medical condition and prescribed medicines files from 2005 to 2015.

In general, longitudinal surveys collect data from the same respondent at multiple points in time. Moreover, the MEPS longitudinal data files have variables that allow tracking changes in characteristics and health status for specific respondents over the two years of follow-up. Four of these variables represent baseline and follow-up data for HRQoL and the use of the MEPS longitudinal data file will make evaluating the change in the HRQoL possible.

### Subjects

The study included all noninstitutionalized U.S. adults (≥18 years) who had depression documented in their medical condition files during the first year of the two-year follow-up. Subjects were included if they completed a two-year of follow-up in MEPS with a final person weight greater than zero to be representative of the national population. Subjects were excluded if they have missing data for the HRQoL or if they died during the two-year follow-up, to avoid the impact of the zero result for the HRQoL in the follow-up variable.

### Data extraction

**Fig 1:**
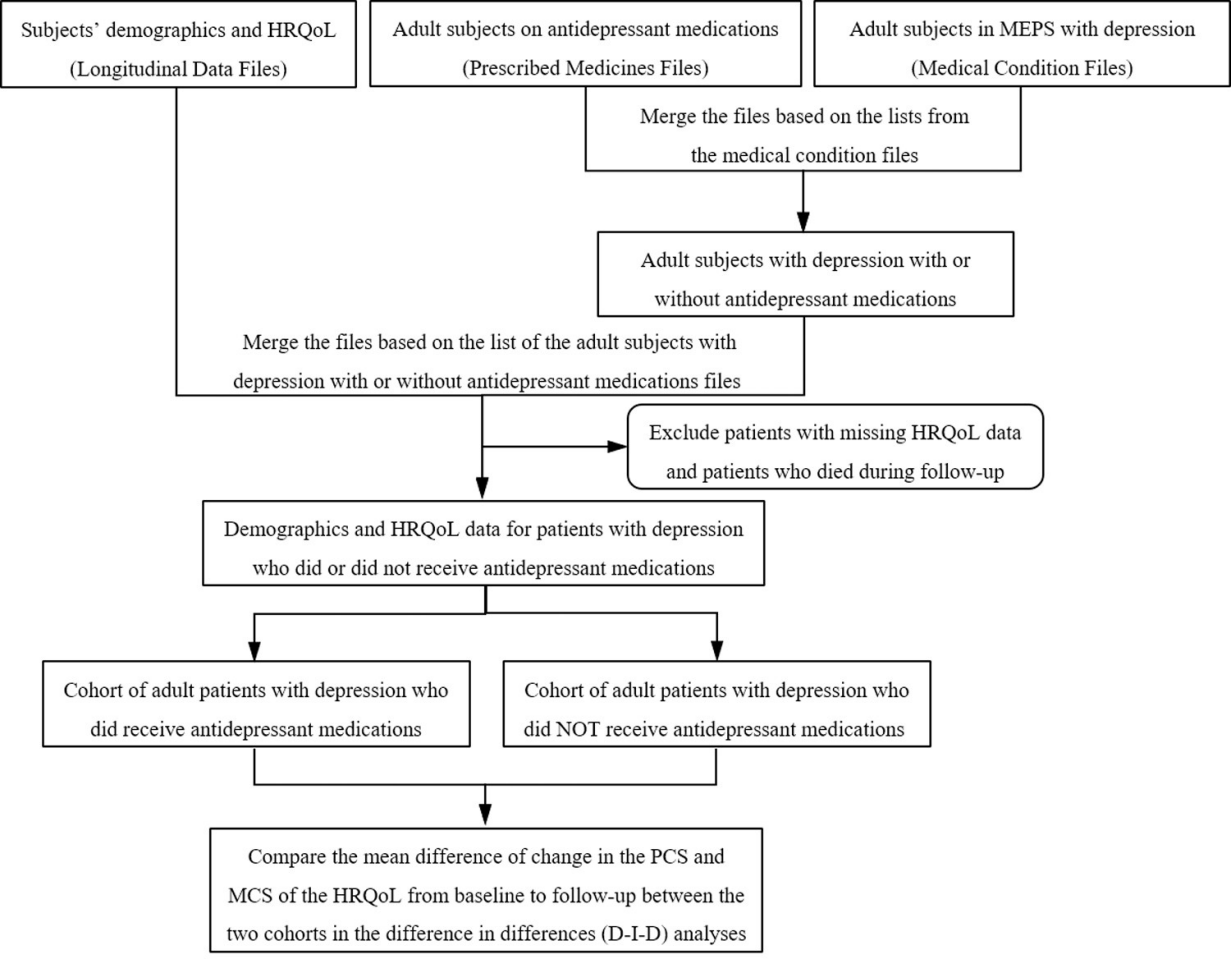
The flow diagram for the design of the study. Abbreviations: HRQoL: Health related quality of life, MEPS: Medical Expenditure Panel Survey, PCS: Physical component summary, MCS: Mental component summary, D-I-D: Difference in differences.

The MEPS medical condition files for the years 2005–2015 were analyzed and adult patients with a diagnosis of depression disorder were identified using the International Statistical Classification of Diseases, Ninth Revision, Clinical Modification (ICD-9 CM) codes of 296.xx or 311.xx which were previously recognized as appropriate identifier for patients with depression in MEPS [25–28]. The subset of patients with depression from the 11 medical condition files were then merged with the associated prescribed medicines data files from 2005–2015 to identify depression patients who utilized any antidepressant medication during the follow-up. The Multum drug database was used to identify antidepressant medications and the TC1 code of 242 and TC1S1 code of 249 were used to identify the use of any antidepressant medications in the prescribed medicines data files [26]. Consecutively, patients with depression disorder were separated into two groups based on the use of any antidepressant medications. The previously merged medical conditions and prescribed medicines files were then merged with the related longitudinal data files, to include only patients that had two years of follow-up (Fig 1).

The HRQoL in the MEPS is measured using the SF-12 health survey (version 2) and report the physical component summary (PCS) and mental component summary (MCS) [29, 30]. The 12 items on the SF-12 comprising the eight quality of life domains are calculated into two summary measures: the PCS and the MCS. The PCS focuses primarily on physical functioning, role limitations due to physical health problems, bodily pain, general health, and vitality (energy/fatigue). The MCS focuses on social functioning, role limitations due to emotional problems, and mental health (psychological distress and psychological well-being). These two components are represented with four variables in MEPS, two baseline (PCS2 and MCS2) and two follow-up (PCS4 and MCS4) variables. The PCS2 and MCS2 are captured at the beginning of the first year and the PCS4 and MCS4 are captured at the end of the second year of two-year follow-up. The mean differences for the change in the PCS and MCS from baseline to follow-up were used as the main outcomes in the study. The study assessed the significance of the difference between these mean differences in the two cohorts (the users and non-users of antidepressant medications).

### Statistical analysis

Descriptive statistics were used to describe the demographics of the study population and the results were presented as averages for all years included in the study (2005–2015). The independent *t* and *χ*^*2*^ tests were used to compare the demographical characteristics between the two cohorts in the study. The outcomes were presented as the mean for each component at both levels followed by the mean difference of change for the overall sample, then for each cohort separately.

The difference in differences (D-I-D) analysis was used to assess the significance of the change between the two cohorts [31]. D-I-D analysis has been used in the analysis of our study for several reasons that makes it more appropriate and accurate for our objective than other methods that can be used. These reasons include that our population contains a diverse cohort of patients that were compared to their baseline levels in terms of within group change over time to limit the effect of the individual differences on the overall change from baseline for each cohort. Whereas the comparison between the two cohorts is mainly based on the variation in the mean differences of change for each cohort. Thus, it is an advantage that makes the D-I-D analysis useful in the settings where randomization is not possible. Moreover, the D-I-D analysis is intended to attenuate the influence of baseline demographical and socioeconomical characteristics and selection bias on the intended outcomes [32, 33]. Several studies have used this technique to study the effect of a specific intervention on large scale over a specific period of time and were deemed appropriate [34–36]. The D-I-D was carried on as a univariate analysis for the overall combined sample from 11 panels in the basic analysis, then data from each individual panel was analyzed individually to test the consistency of the findings over the years. Moreover, the robustness of the results from the univariate analyses, for the combined and individual panels, were assessed in multivariate analyses to adjust for the effect of all other baseline demographical and socioeconomical variables in the study.

The D-I-D was conducted using the SAS PROC GLM function [31]. The analyses for the demographical variables in the study utilized SAS PROC SURVEY function and incorporated sample weights and standard errors to adjust for the complex sampling design. The α < 0.05 level was used for statistical significance. All data management and analyses described in this paper were performed using Statistical Analysis System (SAS) software, version 9.4 (SAS Institute Inc., 2014).

## Results

### Patient characteristics

Over the duration of the study (2005–2016), on average there were 17.47 million adult patients diagnosed with depression disorder every year with two-year follow up. About 57.6% of these patients received treatment with antidepressant medications. The average age for included adult patients with depression was 48.3 years; patients receiving treatment with antidepressant medications were slightly older than patients that were not (49.2 vs. 47.1 years, *p*-value<0.0001). Females accounted for more than two-thirds of our sample (67.9%); and 60.6% of them received antidepressants compared to 51.5% of their male counterparts (*p*-value<0.0001). The majority were White (88.9%) and those were more likely to receive antidepressant medications (59.1%) compared to other races (Table 1). Compared to Hispanic subjects, non-Hispanic were more likely to receive antidepressants (52.4% vs. 58.5%, *p*-value<0.0001).

**Table 1:** Average weighted numbers and proportions for baseline demographical and socioeconomical variables.

Variable		Received antidepressant medication		
	Overall	Yes	No	p-value*
	N = 17,472,864	n = 10,071,920	n = 7,400,944	
**Age, mean [95%CI]**	**48.33 [47.8–48.8]**	**49.2 [48.6–49.9]**	**47.1 [46.3–47.9]**	**<0.0001**
**Gender**				**<0.0001**
**Male**	**5,605,090 (32.1)**	**2,885,603 (28.7)**	**2,719,487 (36.7)**	
**Female**	**11,867,774 (67.9)**	**7,186,317 (71.3)**	**4,681,457 (63.3)**	
**Race**				**<0.0001**
**White**	**15,525,826 (88.9)**	**9,171,012 (91.0)**	**6,354,814 (85.9)**	
**Black**	**1,124,334 (6.4)**	**512,983 (5.1)**	**611,351 (8.2)**	
**American Indian/Alaska Native**	**163,958 (0.9)**	**89,687 (0.9)**	**74,271 (1.0)**	
**Asian/Native Hawaiian/Pacific Islander**	**272,371 (1.6)**	**97,924 (1.0)**	**174,447 (2.4)**	
**Multiple races reported**	**386,375 (2.2)**	**200,316 (2.0)**	**186,059 (2.5)**	
**Ethnicity**				**<0.0001**
**Hispanic**	**1,386,966 (7.9)**	**727,027 (6.6)**	**659,939 (9.8)**	
**Non-Hispanic**	**16,085,898 (92.1)**	**9,411,980 (93.4)**	**6,673,918 (90.2)**	
**Marital status**				**<0.0001**
**Married**	**8,319,982 (47.6)**	**5,077,676 (50.5)**	**3,242,306 (43.8)**	
**Widowed**	**1,322,959 (7.6)**	**778,923 (7.7)**	**544,036 (7.4)**	
**Divorced**	**3,131,258 (17.9)**	**1,861,016 (18.5)**	**1,270,242 (17.2)**	
**Separated**	**630,804 (3.6)**	**386,037 (3.8)**	**244,767 (3.3)**	
**Never married**	**4,067,861 (23.3)**	**1,968,268 (19.5)**	**2,099,593 (28.3)**	
**Family income level**				**0.0216**
**Poor/Negative**	**3,051,969 (17.5)**	**1,684,179 (16.7)**	**1,367,790 (18.5)**	
**Near Poor**	**965,430 (5.5)**	**563,886 (5.6)**	**401,544 (5.4)**	
**Low income**	**2,470,225 (14.1)**	**1,359,592 (13.5)**	**1,110,633 (15.0)**	
**Middle income**	**5,181,983 (29.7)**	**2,955,985 (29.4)**	**2,225,998 (30.1)**	
**High Income**	**5,803,256 (33.2)**	**3,508,278 (34.8)**	**2,294,978 (31.0)**	
**Insurance coverage**				**<0.0001**
**Private insurance**	**11,252,415 (64.4)**	**6,621,627 (65.8)**	**4,630,788 (62.6)**	
**Public insurance**	**4,728,751 (27.1)**	**2,740,793 (27.2)**	**1,987,958 (26.9)**	
**Uninsured**	**1,491,698 (8.5)**	**709,500 (7.0)**	**782,198 (10.5)**	

Results are presented as average of frequency and (%) from 11 panels, unless otherwise indicated.

* *p-*value <0.05 was considered statistically significant and numbers in bold indicate significant results.

Married patients represented the largest proportion of the study sample (47.6%) followed by patients who have never been married (23.3%), and the latter were the least likely to receive antidepressants compared to others. The majority of patients (62.9%) came from middle- and high-income households. Furthermore, most patients (64.4%) were privately insured and uninsured subjects were less likely to receive treatment with antidepressant medications (47.6%) compared to the privately (58.8%) or publicly (58.0%) insured counterparts. Patients’ baseline demographics and their distribution between the two cohorts based on the antidepressant medication used is depicted in Table 1. In addition, patients on antidepressant medications were more likely to have a lower baseline levels on the PCS and MCS as seen in Tables 2–4, S1 and S2 Tables.

**Table 2:** Least square means and mean difference of change on the physical and mental component summary of the HRQoL for the combined panels analysis (2005–2015) and the significance of the difference from the uni- and multivariate D-I-D analysis.

	Overall Change			Received antidepressant medication						
				Yes			No			*p*-value*
Year	Baseline	Follow-up	Mean Difference	Baseline	Follow-up	Mean Difference	Baseline	Follow-up	Mean Difference	
**Univariate**										
**PCS**	44.52	44.18	- 0.34	43.58	43.23	- 0.35	45.72	45.38	- 0.34	0.9595
**MCS**	42.08	43.30	1.22	41.03	42.31	1.28	43.41	44.54	1.13	0.5284
**Multivariate**										
**PCS**	44.52	44.18	- 0.34	42.50	41.85	- 0.65	43.86	43.31	- 0.55	0.6405
**MCS**	42.08	43.30	1.22	40.32	41.50	1.18	42.99	43.92	0.93	0.3191

Results were presented as least square means from baseline and follow-up for both groups and mean difference within each group.

Age, gender, race, ethnicity, marital status, poverty level, and insurance coverage were included in the multivariate analysis to adjust for the effect of these significant factors between the cohort on the outcomes’ variables.

* *p-*value <0.05 was considered statistically significant and numbers in bold indicate significant results.

**Table 3:** Least square means and mean difference of change on the physical component summary of the HRQoL (univariate analysis).

	Overall Change			Received antidepressant medication						
				Yes			No			
Year	Baseline	Follow-up	Mean Difference	Baseline	Follow-up	Mean Difference	Baseline	Follow-up	Mean Difference	*p*-value*
**2005**	**43.59**	**42.95**	**- 0.64**	**43.25**	**42.89**	**- 0.36**	**44.05**	**43.05**	**- 1.00**	**0.3418**
**2006**	**44.38**	**44.00**	**- 0.38**	**42.74**	**42.38**	**- 0.36**	**46.58**	**46.17**	**- 0.41**	**0.9297**
**2007**	**45.09**	**44.72**	**- 0.37**	**44.12**	**44.24**	**0.12**	**46.54**	**45.44**	**- 1.10**	**0.1313**
**2008**	**44.12**	**43.57**	**- 0.55**	**43.89**	**43.29**	**- 0.60**	**44.42**	**43.95**	**- 0.47**	**0.8284**
**2009**	**44.40**	**43.81**	**- 0.59**	**43.09**	**42.93**	**- 0.16**	**46.08**	**44.96**	**- 1.12**	**0.1397**
**2010**	**44.29**	**43.71**	**- 0.58**	**43.53**	**42.85**	**- 0.68**	**45.24**	**44.78**	**- 0.46**	**0.7443**
**2011**	**44.35**	**43.99**	**- 0.36**	**43.86**	**43.57**	**- 0.29**	**44.94**	**44.51**	**- 0.43**	**0.8067**
**2012**	**44.02**	**44.08**	**0.06**	**43.19**	**43.15**	**- 0.04**	**45.04**	**45.21**	**0.17**	**0.7235**
**2013**	**44.65**	**44.49**	**- 0.16**	**43.58**	**43.26**	**- 0.32**	**45.87**	**45.90**	**0.03**	**0.5488**
**2014**	**45.13**	**44.95**	**- 0.18**	**43.97**	**43.18**	**- 0.79**	**46.51**	**47.06**	**0.55**	**0.0265**
**2015**	**45.29**	**45.00**	**- 0.29**	**43.91**	**43.65**	**- 0.26**	**46.99**	**46.66**	**- 0.33**	**0.9039**

Results were presented as least square means from baseline and follow-up for both groups and mean difference within each group.

* *p-*value <0.05 was considered statistically significant and numbers in bold indicate significant results.

**Table 4:** Least square means and mean difference of change on the mental component summary of the HRQoL (univariate analysis).

** **	**Overall Change**			**Received antidepressant medication**						
				**Yes**			**No**			** **
**Year**	**Baseline**	**Follow-up**	**Mean Difference**	**Baseline**	**Follow-up**	**Mean Difference**	**Baseline**	**Follow-up**	**Mean Difference**	*p*-value*
**2005**	**42.07**	**42.86**	**0.79**	**40.21**	**41.58**	**1.37**	**44.59**	**44.6**	**0.01**	**0.1109**
**2006**	**41.39**	**42.75**	**1.36**	**39.88**	**41.39**	**1.51**	**43.41**	**44.56**	**1.15**	**0.6742**
**2007**	**41.97**	**43.38**	**1.41**	**40.96**	**42.59**	**1.63**	**43.48**	**44.56**	**1.08**	**0.6029**
**2008**	**41.35**	**42.58**	**1.23**	**40.16**	**41.13**	**0.97**	**42.92**	**44.49**	**1.57**	**0.4824**
**2009**	**41.64**	**43.11**	**1.47**	**40.67**	**41.81**	**1.14**	**42.87**	**44.78**	**1.91**	**0.3659**
**2010**	**41.92**	**42.85**	**0.93**	**40.85**	**41.78**	**0.93**	**43.25**	**44.16**	**0.91**	**0.9862**
**2011**	**41.47**	**42.61**	**1.14**	**40.83**	**41.68**	**0.85**	**42.25**	**43.75**	**1.5**	**0.4005**
**2012**	**42.11**	**43.85**	**1.74**	**41.34**	**42.72**	**1.38**	**43.04**	**45.22**	**2.18**	**0.3022**
**2013**	**42.7**	**43.57**	**0.87**	**42.02**	**43.47**	**1.45**	**43.48**	**43.68**	**0.2**	**0.0894**
**2014**	**43.14**	**44.26**	**1.12**	**42.06**	**43.75**	**1.69**	**44.42**	**44.87**	**0.45**	**0.126**
**2015**	**42.61**	**43.88**	**1.27**	**41.56**	**42.8**	**1.24**	**43.89**	**45.21**	**1.32**	**0.9251**

Results were presented as least square means from baseline and follow-up for both groups and mean difference within each group.

* *p-*value <0.05 was considered statistically significant and numbers in bold indicate significant results.

### D-I-D univariate analyses

When comparing data from the combined panels of 2005 to 2015 for the cohort of patients not using antidepressant medications, the cohort that was using antidepressant medications has shown some improvement on the MCS, but not the PCS of the HRQoL. However, the results from the D-I-D univariate analyses for the difference in the mean differences of change on the PCS and MCS shows no significant difference from baseline to follow-up between the two cohorts of those who received antidepressant medications compared to those who did not (PCS: - 0.35 vs. - 0.34, *p*-value 0.9595; MCS: 1.28 vs. 1.13, *p*-value 0.5284), as summarized in Table 2. Moreover, when each panel were analyzed individually, this result was consistent for 10 out of 11 panels for the PCS and all 11 panels on the MCS. Additionally, the cohorts of patients that were not receiving antidepressant medications had more years with a positive net mean difference of change (improvement) on the PCS compared to the other cohort (3 years vs. 1 year) as seen in Table 3. Whereas the mean net difference of change on the MCS was always positive for both groups as presented in Table 4.

### D-I-D multivariate analyses

The differences between the two cohorts in the baseline demographical and socioeconomic variables (presented in Table 1) are significant confounders that might affect our results. Therefore, we carried the D-I-D multivariate analyses for the combined and individual panels to adjust for the effect of these variables on the mean differences of change and to ensure the robustness of the results. The results from the D-I-D multivariate sensitivity analyses controlling for the effect of age, gender, race, ethnicity, marital status, poverty level, and insurance coverage confirmed the findings from the overall univariate analyses for the combined data from panels 2005 to 2015 (PCS: - 0.65 vs. - 0.55, *p*-value 0.6405; MCS: 1.18 vs. 0.93, *p*-value 0.3191), as depicted in Table 2. Moreover, when each panel were analyzed individually to assess the robustness of the results from the overall multivariate analysis and from the univariate analyses, this result was consistent for 10 out of 11 panels for both outcomes, PCS and MCS. The results from the multivariate analyses for each year were presented in S1 and S2 Tables.

## Discussion

It is generally well known that depression disorder has a significant impact on the HRQoL of patients. This comparative cohort, secondary database analysis was conducted to examine the effect of pharmacological treatment with antidepressant medications on the HRQoL in adult patients with depression. On average for the 11 years that were included in the analysis, about 17.5 million of the non-institutionalized US adults are diagnosed with depression disorder every year. Around 58% of the overall combined cohort received antidepressant medications and the use of these medications was associated with more positive change on the MCS but not the PCS of the HRQoL. However, this change on the MCS did not reach significance when compared to the cohort of patients that were not using antidepressant medications. Although there were some significant differences between the two cohorts in the baseline demographical and socioeconomical characteristics, such as age, gender, race, and poverty level, the findings from the univariate analyses were robust after adjustment for differences in these characteristics in the multivariate analyses for the overall combined panels analysis and in almost all included individual panels for both PCS and MCS.

The results indicate that in the US more women in general are diagnosed with depression disorder than men (67.9% vs. 32.1%, respectively). That is in line with a report published by the World Health Organization (WHO) which shows that the depression disorder impacts more women than men [37]. In a data brief using nationally representative sample, the Center for Disease Control and Prevention (CDC) reported that serious psychological illnesses are impacting more women than men in the US [38]. The reason behind this variability in the prevalence of mental illnesses between men and women is not fully understood, however, there are some theories that tried to explain it. Gender bias is one of these theories and it suggests that physicians are more likely to diagnose depression in and prescribe antidepressants medication to women compared to men even if both had similar scores on standardized measures for depression. Moreover, disclosing mental health issues and seeking help from primary health care providers is more common among women, as compared to men who usually seek help from more specialized mental health providers and need more inpatient care [37].

Many randomized control trials (RCTs) evaluated the effect of psychotherapy and pharmacotherapy on patient’s quality of life. Forty four RCTs were included in a meta-analysis that was set to examine the effects of psychotherapy on the quality of life; it found that psychotherapy improves various domains of patient’s life and the largest effect was observed in the mental health component whereas the effect was limited on the physical health component [39]. Another larger meta-analysis including one hundred and fifty-three RCTs show that the combination of both psychotherapy and pharmacotherapy was significantly improving patient’s quality of life compared to either of the treatments alone; in general, psychotherapy had marginally better effect compared to pharmacotherapy [15]. After adjusting for the effect of publication bias in this meta-analysis, the subgroup analysis uncovered a difference between short-term trials (treatment duration of ≤ 3 months) which found an insignificantly better result for pharmacotherapy and long-term trials (treatment duration of > 3 months) which shows insignificantly superior result for psychotherapy [15]. In addition, the adjuvant use of atypical antipsychotic medication was found to be associated with a lower quality of life compared with the use of antidepressant medications alone [40].

Two cohort studies evaluating the effect of initiating pharmacotherapy on the HRQoL among patients with MDD found that the improvement on the HRQoL measures was limited to the first two to three months after the initiation of pharmacotherapy and the long-term effect on the HRQoL has never reached the scores for the general population [41, 42]. The finding from our study support the finding of these two cohort studies. Our study was designed and conducted to include all patients with depression regardless of their initial diagnosis date, which is not available in the database. No improvement was seen in our long-term follow-up (more than one year) with the use of antidepressant medications on either component of the HRQoL.

Regardless of the symptoms associated with depression and the extent of their impact on these patient’s quality of life and the side effects of antidepressant medications, it is worth to re-evaluate the effectiveness and the placement of psychotherapy in these patients. A review article that included three meta-analyses was intended for comparison between placebo, psychotherapy, and antidepressants. The review showed that there was no statistically significant difference between placebo treatment and psychotherapy; besides that, patients who were receiving either placebo or psychotherapy showed lower relapse rate than those who were on antidepressant medications [43]. These results, to some extent, coincide with the results of this study as the use of antidepressant medications was associated with higher rate of relapse compared to placebo, which makes the continuous prescribing of antidepressant medications a matter of preference rather than a necessity. Taking this limitation into consideration, it is necessary to reconsider the importance of non-pharmacological therapy, including psychotherapy, and its placement in the clinical practice guideline. Physicians, mainly primary care providers who are caring for most of these patients, may need to reconsider referring patients with depression to receive some kind of non-pharmacological therapy, such as behavioral therapy, psychotherapy, social support sessions, or education before or when initiating these patients on antidepressant medications; mainly since there was no persisting impact for these medications on the patients’ HRQoL.

Like other retrospective observational studies using secondary databases, our study had some limitations. The use of retrospective data precludes the ability to make a causal relationship; thus, we cannot exclude that the use, or lack of use, of antidepressant medications can affect the HRQoL measures. However, this finding indicates that future research evaluating the benefits of psychopharmacotherapy should include the HRQoL as one of the outcomes as it represents patients’ perspective. The study included all patients that had a documented diagnosis for depression regardless of the time for the first diagnosis. This limits our ability to compare between patients with long-term diagnosis for depression and the newly diagnosed patients. The data in the medical condition files in MEPS does not provide details that allow for the specific diagnosis of each type of depression, to limit the recognition of these patients. Therefore, it is not possible to compare the validity of the results among patients with subtypes of depression. The ICD-9 codes that were used to identify depression is not limited to depression per say, it rather includes all patients with mood disorders. However, these codes were confirmed to provide good estimate for patients with depression as at least 90% of these patients with mood disorder are essentially diagnosed with depression [26]. The study used the MEPS longitudinal data files which follow included subjects for two years but patients that were diagnosed with depression at the end of the first year, or later, were not included to allow for the comparison between baseline and follow-up variables for HRQoL. This may underestimate the number of patients living with depression disorder in the US and the utilization for antidepressant medications, which will make our numbers varies from other studies that used the cross-sectional data files. However, this should not affect our finding about the relationship between the use of antidepressant medications and the HRQoL. Lastly, the MEPS does not provide information on the severity of depression which limit our ability to control for the effect of this important factor. However, the D-I-D analysis compare each subject’s follow-up levels to his/her individual baseline levels for the PCS and MCS and investigate the overall change for the group which should minimize the impact of this factor on the overall analysis.

## Conclusion

The ultimate goal of using antidepressant medications or psychotherapy is to improve patients’ important outcomes, such as HRQoL. The real-world effect of using antidepressant medications does not continue to improve patients’ HRQoL over time, as the change in HRQoL was comparable to patients who did not use any antidepressant medications. Future studies should not focus on the use of pharmacotherapy only, it should rather investigate the long-term impact of pharmacological and non-pharmacological interventions, such as behavioral therapy, psychotherapy, social support sessions, education, or combined interventions, on these patients’ HRQoL.

## Supporting information

S1 Table Least square means and mean difference of change on the physical component summary of the HRQoL (multivaraite analysis). (DOCX)

S2 Table Least square means and mean difference of change on the mental component summary of the HRQoL (multivaraite analysis). (DOCX)

S1 File Links to the data files on MEPS database. (DOCX)
