# Supplementary material for: Antidepressants and health-related quality of life (HRQoL) for patients with depression: Analysis of the medical expenditure panel survey from the United States
Source: PLoS One. 2022 Apr 20;17(4):e0265928. doi: 10.1371/journal.pone.0265928 (PMC9020683; doi:10.1371/journal.pone.0265928)
Supplement: S2 Table — (DOCX) [file pone.0265928.s002.docx]

| **S2 Table.** Least square means and mean difference of change on the mental component summary of the HRQoL (Multivaraite analysis) | | | | | | | | | | |
| --- | --- | --- | --- | --- | --- | --- | --- | --- | --- | --- |
|  | **Overall Change** | | | **Received antidepressant medication** | | | | | | |
|  |  |  |  | **Yes** | | | **No** | | | ***p*-values^*^** |
| **Year** | **Baseline** | **Follow-up** | **Mean Difference** | **Baseline** | **Follow-up** | **Mean Difference** | **Baseline** | **Follow-up** | **Mean Difference** |  |
| **2005** | 42.07 | 42.86 | 0.79 | 38.72 | 38.10 | -0.62 | 43.32 | 41.59 | -1.73 | 0.2338 |
| **2006** | 41.39 | 42.75 | 1.36 | 38.54 | 39.84 | 1.3 | 42.32 | 42.89 | 0.57 | 0.4506 |
| **2007** | 41.97 | 43.38 | 1.41 | 38.34 | 40.81 | 2.47 | 41.83 | 42.72 | 0.89 | 0.1923 |
| **2008** | 41.35 | 42.58 | 1.23 | 39.87 | 39.44 | -0.43 | 42.18 | 42.30 | 0.12 | 0.5547 |
| **2009** | 41.64 | 43.11 | 1.47 | 40.14 | 41.44 | 1.3 | 42.60 | 44.67 | 2.07 | 0.4060 |
| **2010** | 41.92 | 42.85 | 0.93 | 41.60 | 42.68 | 1.08 | 43.77 | 45.24 | 1.47 | 0.7064 |
| **2011** | 41.47 | 42.61 | 1.14 | 40.93 | 40.93 | 0 | 42.63 | 43.37 | 0.74 | 0.3457 |
| **2012** | 42.11 | 43.85 | 1.74 | 40.62 | 42.93 | 2.31 | 43.28 | 46.03 | 2.75 | 0.6032 |
| **2013** | 42.70 | 43.57 | 0.87 | 40.93 | 43.79 | 2.86 | 43.61 | 44.77 | 1.16 | **0.0314** |
| **2014** | 43.14 | 44.26 | 1.12 | 41.70 | 42.76 | 1.06 | 43.87 | 43.65 | -0.22 | 0.1344 |
| **2015** | 42.61 | 43.88 | 1.27 | 40.66 | 42.12 | 1.46 | 43.89 | 45.11 | 1.22 | 0.7448 |
| Results were presented as least square means from baseline and follow-up for both groups and mean difference within each group.  Age, gender, race, ethnicity, marital status, poverty level, and insurance coverage were included in the multivariate analysis to adjust for the effect of these significant factors between the cohort on the outcomes’ variables.  ^*^ *p-*value <0.05 was considered statistically significant and numbers in bold indicate significant results. | | | | | | | | | | |
