# Supplementary material for: Antidepressants and health-related quality of life (HRQoL) for patients with depression: Analysis of the medical expenditure panel survey from the United States
Source: PLoS One. 2022 Apr 20;17(4):e0265928. doi: 10.1371/journal.pone.0265928 (PMC9020683; doi:10.1371/journal.pone.0265928)
Supplement: S1 File — (DOCX) [file pone.0265928.s003.docx]

| **Number** | **Link to the data file** | **Description of the data file** | **Year of the data file** |
| --- | --- | --- | --- |
| 1 | [HC-183](https://meps.ahrq.gov/data_stats/download_data_files_detail.jsp?cboPufNumber=HC-183) | MEPS Panel 19 Longitudinal Data File | 2014-2015 |
| 2 | [HC-180](https://meps.ahrq.gov/data_stats/download_data_files_detail.jsp?cboPufNumber=HC-180) | 2015 Medical Conditions File | 2015 |
| 3 | [HC-178A](https://meps.ahrq.gov/data_stats/download_data_files_detail.jsp?cboPufNumber=HC-178A) | 2015 Prescribed Medicines File | 2015 |
| 4 | [HC-172](https://meps.ahrq.gov/data_stats/download_data_files_detail.jsp?cboPufNumber=HC-172) | MEPS Panel 18 Longitudinal Data File | 2013-2014 |
| 5 | [HC-170](https://meps.ahrq.gov/data_stats/download_data_files_detail.jsp?cboPufNumber=HC-170) | 2014 Medical Conditions File | 2014 |
| 6 | [HC-168A](https://meps.ahrq.gov/data_stats/download_data_files_detail.jsp?cboPufNumber=HC-168A) | 2014 Prescribed Medicines File | 2014 |
| 7 | [HC-164](https://meps.ahrq.gov/data_stats/download_data_files_detail.jsp?cboPufNumber=HC-164) | MEPS Panel 17 Longitudinal Data File | 2012-2013 |
| 8 | [HC-162](https://meps.ahrq.gov/data_stats/download_data_files_detail.jsp?cboPufNumber=HC-162) | 2013 Medical Conditions File | 2013 |
| 9 | [HC-160A](https://meps.ahrq.gov/data_stats/download_data_files_detail.jsp?cboPufNumber=HC-160A) | 2013 Prescribed Medicines File | 2013 |
| 10 | [HC-156](https://meps.ahrq.gov/data_stats/download_data_files_detail.jsp?cboPufNumber=HC-156) | MEPS Panel 16 Longitudinal Data File | 2011-2012 |
| 11 | [HC-154](https://meps.ahrq.gov/data_stats/download_data_files_detail.jsp?cboPufNumber=HC-154) | 2012 Medical Conditions File | 2012 |
| 12 | [HC-152A](https://meps.ahrq.gov/data_stats/download_data_files_detail.jsp?cboPufNumber=HC-152A) | 2012 Prescribed Medicines File | 2012 |
| 13 | [HC-148](https://meps.ahrq.gov/data_stats/download_data_files_detail.jsp?cboPufNumber=HC-148) | MEPS Panel 15 Longitudinal Data File | 2010-2011 |
| 14 | [HC-146](https://meps.ahrq.gov/data_stats/download_data_files_detail.jsp?cboPufNumber=HC-146) | 2011 Medical Conditions File | 2011 |
| 15 | [HC-144A](https://meps.ahrq.gov/data_stats/download_data_files_detail.jsp?cboPufNumber=HC-144A) | 2011 Prescribed Medicines File | 2011 |
| 16 | [HC-139](https://meps.ahrq.gov/data_stats/download_data_files_detail.jsp?cboPufNumber=HC-139) | MEPS Panel 14 Longitudinal Data File | 2009-2010 |
| 17 | [HC-137](https://meps.ahrq.gov/data_stats/download_data_files_detail.jsp?cboPufNumber=HC-137) | 2010 Medical Conditions File | 2010 |
| 18 | [HC-135A](https://meps.ahrq.gov/data_stats/download_data_files_detail.jsp?cboPufNumber=HC-135A) | 2010 Prescribed Medicines File | 2010 |
| 19 | [HC-130](https://meps.ahrq.gov/data_stats/download_data_files_detail.jsp?cboPufNumber=HC-130) | MEPS Panel 13 Longitudinal Data File | 2008-2009 |
| 20 | [HC-128](https://meps.ahrq.gov/data_stats/download_data_files_detail.jsp?cboPufNumber=HC-128) | 2009 Medical Conditions File | 2009 |
| 21 | [HC-126A](https://meps.ahrq.gov/data_stats/download_data_files_detail.jsp?cboPufNumber=HC-126A) | 2009 Prescribed Medicines File | 2009 |
| 22 | [HC-122](https://meps.ahrq.gov/data_stats/download_data_files_detail.jsp?cboPufNumber=HC-122) | MEPS Panel 12 Longitudinal Data File | 2007-2008 |
| 23 | [HC-120](https://meps.ahrq.gov/data_stats/download_data_files_detail.jsp?cboPufNumber=HC-120) | 2008 Medical Conditions File | 2008 |
| 24 | [HC-118A](https://meps.ahrq.gov/data_stats/download_data_files_detail.jsp?cboPufNumber=HC-118A) | 2008 Prescribed Medicines File | 2008 |
| 25 | [HC-114](https://meps.ahrq.gov/data_stats/download_data_files_detail.jsp?cboPufNumber=HC-114) | MEPS Panel 11 Longitudinal Data File | 2006-2007 |
| 26 | [HC-112](https://meps.ahrq.gov/data_stats/download_data_files_detail.jsp?cboPufNumber=HC-112) | 2007 Medical Conditions File | 2007 |
| 27 | [HC-110A](https://meps.ahrq.gov/data_stats/download_data_files_detail.jsp?cboPufNumber=HC-110A) | 2007 Prescribed Medicines File | 2007 |
| 28 | [HC-106](https://meps.ahrq.gov/data_stats/download_data_files_detail.jsp?cboPufNumber=HC-106) | MEPS Panel 10 Longitudinal Data File | 2005-2006 |
| 29 | [HC-104](https://meps.ahrq.gov/data_stats/download_data_files_detail.jsp?cboPufNumber=HC-104) | 2006 Medical Conditions File | 2006 |
| 30 | [HC-102A](https://meps.ahrq.gov/data_stats/download_data_files_detail.jsp?cboPufNumber=HC-102A) | 2006 Prescribed Medicines File | 2006 |
| 31 | [HC-098](https://meps.ahrq.gov/data_stats/download_data_files_detail.jsp?cboPufNumber=HC-098) | MEPS Panel 9 Longitudinal Data File | 2004-2005 |
| 32 | [HC-096](https://meps.ahrq.gov/data_stats/download_data_files_detail.jsp?cboPufNumber=HC-096) | 2005 Medical Conditions File | 2005 |
| 33 | [HC-094A](https://meps.ahrq.gov/data_stats/download_data_files_detail.jsp?cboPufNumber=HC-094A) | 2005 Prescribed Medicines File | 2005 |

**Note:** The links contain all related information to the data files; including documentation, codebook, and programming files for SAS, SPSS and STATA, along with the data files in different formats.
